# Comparative Analysis of Osteogenic/Chondrogenic Differentiation Potential in Primary Limb Bud-Derived and C3H10T1/2 Cell Line-Based Mouse Micromass Cultures

**DOI:** 10.3390/ijms140816141

**Published:** 2013-08-05

**Authors:** Roland Takács, Csaba Matta, Csilla Somogyi, Tamás Juhász, Róza Zákány

**Affiliations:** Department of Anatomy, Histology and Embryology, Medical and Health Science Centre, University of Debrecen, Nagyerdei krt. 98, H-4032 Debrecen, Hungary; E-Mails: takacs.roland@med.unideb.hu (R.T.); matta.csaba@med.unideb.hu (C.M.); somogyics@anat.med.unideb.hu (C.S.); juhaszt@anat.med.unideb.hu (T.J.)

**Keywords:** chondrogenesis, osteogenesis, adipogenesis, pluripotency factors, marker gene expression, C3H10T1/2, high density culture

## Abstract

Murine micromass models have been extensively applied to study chondrogenesis and osteogenesis to elucidate pathways of endochondral bone formation. Here we provide a detailed comparative analysis of the differentiation potential of micromass cultures established from either BMP-2 overexpressing C3H10T1/2 cells or mouse embryonic limb bud-derived chondroprogenitor cells, using micromass cultures from untransfected C3H10T1/2 cells as controls. Although the BMP-2 overexpressing C3H10T1/2 cells failed to form chondrogenic nodules, cells of both models expressed mRNA transcripts for major cartilage-specific marker genes including *Sox9*, *Acan*, *Col2a1*, *Snorc*, and *Hapln1* at similar temporal sequence, while notable *lubricin* expression was only detected in primary cultures. Furthermore, mRNA transcripts for markers of osteogenic differentiation including *Runx2*, *Osterix*, *alkaline phosphatase*, *osteopontin* and *osteocalcin* were detected in both models, along with matrix calcification. Although the adipogenic lineage-specific marker gene *FABP4* was also expressed in micromass cultures, Oil Red O-positive cells along with *PPARγ2* transcripts were only detected in C3H10T1/2-derived micromass cultures. Apart from lineage-specific marker genes, pluripotency factors (*Nanog* and *Sox2*) were also expressed in these models, reflecting on the presence of various mesenchymal lineages as well as undifferentiated cells. This cellular heterogeneity has to be taken into consideration for the interpretation of data obtained by using these models.

## 1. Introduction

The main events marking the formation of the embryonic skeleton are cell proliferation and condensation of undifferentiated mesenchymal cells, followed by differentiation into chondroblasts and chondrocytes that can undergo hypertrophy and contribute to tissue calcification, and eventually endochondral bone formation. This process largely depends on complex signalling that is facilitated through cell-cell and cell-matrix contacts, regulated by several adhesion molecules and extracellular components such as N-cadherin and N-CAM, as well as gap junctions [1,2]. It has been widely established that soluble factors including members of the transforming growth factor-β (TGF-β) superfamily, bone morphogenetic proteins (BMPs; BMP-2 in particular) and growth and differentiation factor-5 (GDF-5) play important roles in inducing chondrocyte-specific genes as they are known to upregulate type II collagen expression in mesenchymal cells [3]. Since abnormal regulation of this intricate process may lead to various conditions that affect cartilage and bone [4], studies aimed at elucidating the molecular steps of chondrogenesis and subsequent endochondral bone formation are of particular importance.

Over the past decades, many *in vitro* model systems have been established and validated to study chondrogenesis and early phases of matrix calcification. Since the initial condensation of mesenchymal cells is a prerequisite to their subsequent differentiation, by mimicking these conditions *in vitro*, chondrogenesis can easily be studied. In this manner, high cellular density (HD) favours the formation of cellular interactions, and thus facilitates the chondrogenic differentiation of pluripotent mesenchymal cells. A well-known and easily reproducible avian experimental model to study hyaline cartilage formation *in vitro* was first described by Ahrens and colleagues [5]. In these high density cell cultures (HDC), the inherent capability of chicken limb bud-derived chondroprogenitor mesenchymal cells to spontaneously differentiate to chondroblasts and chondrocytes on days 2 and 3 of culturing is exploited; a well-detectable amount of hyaline cartilage extracellular matrix (ECM) is produced by day 6. A significant advantage of this method over others is its cost-effectiveness and the relative ease by which sufficient amounts of cells can be yielded from embryos at the same developmental stage (Hamburger-Hamilton developmental stages 22–24) by synchronised incubation of fertilised eggs. However, although the main steps of chondro- and osteogenesis are largely conserved during the evolution of vertebrates, there is evidence that certain key signalling pathways are differentially regulated in the avian system; while the extracellular signal-related kinase ERK1/2, member of the mitogen-activated protein kinases (MAPKs), is a negative regulator of chondrogenesis in chicken limb bud-derived HDC [6], ERK-inhibition leads to decreased Sox9 levels in murine chondrocytes [7]. Conversely, ERK1/2 is a positive regulator of chondrogenesis in BMP-2 induced C3H10T1/2 cultures [8]. Moreover, applications of the avian model are also restricted by the limited number of available antisera and published nucleotide sequences. Nonetheless, the significance of such avian models is underpinned by the fact that many basic processes of chondrogenesis were identified using this system [9–14].

Therefore, there is a need for mammalian models to overcome the limitations encountered for the avian system. Mouse embryonic limb bud-derived micromass cultures [15] certainly represent an option; however, they also exhibit certain disadvantages, such as the need of precisely timed pregnancies of multiple female mice to yield the required amount of chondrogenic cells; and the relatively high level of variations between experiments—an inherent feature of primary cell cultures. Nevertheless, one of the key merits of such primary systems is the possibility of using cells derived from transgenic and knockout animals. Mature chondrocytes isolated from articular or other cartilage using mild enzymatic digestion can also be applied with certain restrictions because chondrocytes deprived of their ECM rapidly lose their characteristic phenotype and tend to dedifferentiate under *in vitro* conditions owing to lack of physiological stimuli [16].

As an attempt to overcome such limitations posed by primary cultures, various cell lines with chondrogenic and osteogenic capabilities have been established over the past decades. Examples include the ATDC5 cell line originally isolated from a differentiating culture of murine AT805 teratocarcinoma [17]; RCJ 3.1, a clonally derived cell population isolated from 21-day foetal rat calvaria [18]; or the murine embryonic multipotential mesenchymal cell line C3H10T1/2 [2]. Micromass cultures established from C3H10T1/2 cells are an attractive system to study *in vitro* chondrogenesis because these cells do not spontaneously differentiate under normal culture conditions. This, at the same time, is also a disadvantage because it necessitates administration of exogenous factors into the culture medium, such as BMP-2 or TGF-β [2,19]. To address this constraint, a plasmid containing the human BMP-2 has been transfected into C3H10T1/2 cells and the constitutive expression of this morphogen, as an autocrine-paracrine factor, drives *in vitro* chondrogenesis of this cell clone [20].

Although transition from using animal models to human cell line or mesenchymal stem cell (hMSC) based systems to study *in vitro* chondrogenesis is inevitable, many laboratories are still using these cost-effective and simple animal systems for various reasons; novel therapeutic targets for most of the diseases that affect the musculoskeletal system (*i.e.*, osteoarthritis) are easier to identify using such models, and also because before clinical trials are initiated, the safety profile and efficacy of drug candidates are usually tested in these animal models. Although chondrogenic differentiation of the C3H10T1/2 cell line and primary limb bud-derived micromass cultures has been extensively studied, no comprehensive report on the mRNA expression profiles of genes that mark differentiation towards various mesenchymal (*i.e.*, chondro-, osteo- and adipogenic) lineages in these two murine models under identical HD culturing conditions has been published. In this study, therefore, by monitoring an array of marker genes we provide a detailed analysis of the differentiation potential of BMP-2 induced C3H10T1/2 micromass cultures and mouse embryonic limb bud-derived micromass cultures, with special emphasis on the osteo- and chondrogenic differentiation of these systems, to enable easier comparison of their differentiation potentials.

## 2. Results

### 2.1. BMP-2 Overexpressing C3H10T1/2-Derived and Embryonic Limb Bud-Derived Micromass Cultures Show Different Morphology

**Figure 1:**
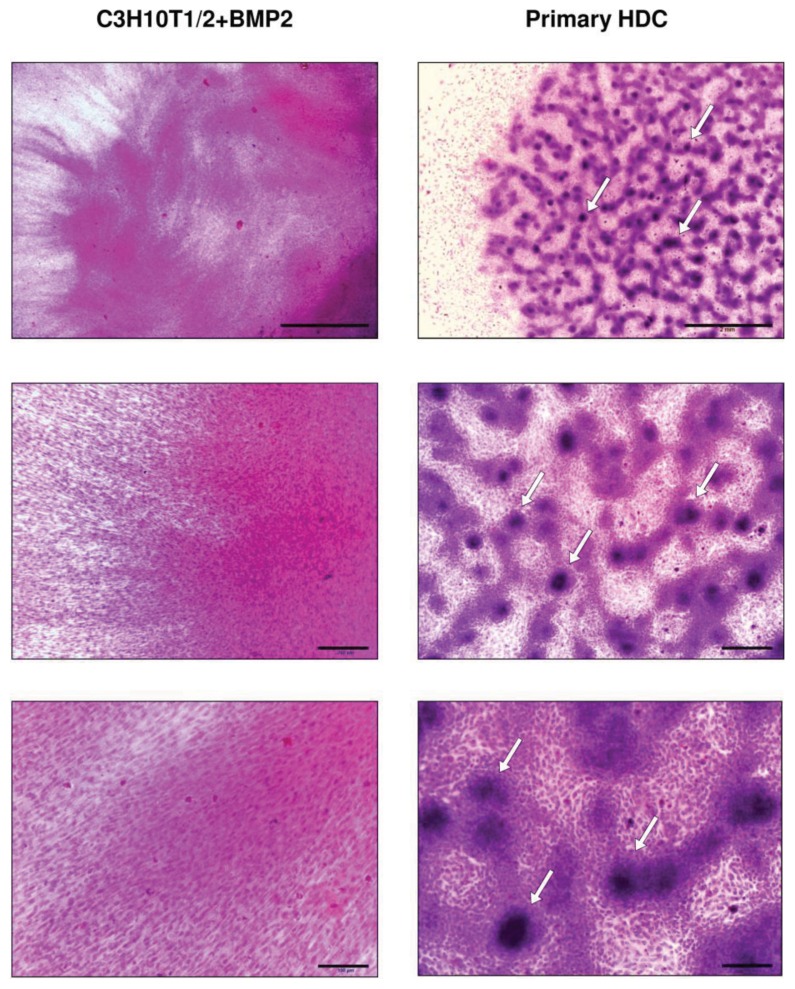
Morphology of 3-day-old micromass cultures established from BMP-2 overexpressing C3H10T1/2 and embryonic limb bud-derived mesenchymal cells after staining with HE. Original magnification was 2× (upper panels), 10× (middle panels), and 20× (lower panels). Scale bars: 2 mm (upper panels), 200 μm (middle panels), and 100 μm (lower panels). In the upper panels, both the peripheral (left) and central (right) regions of micromass cultures are shown. Arrows point at precartilaginous nodules in primary embryonic limb bud-derived HDC. Representative photomicrographs of 3 independent experiments are shown.

**Figure 2:**
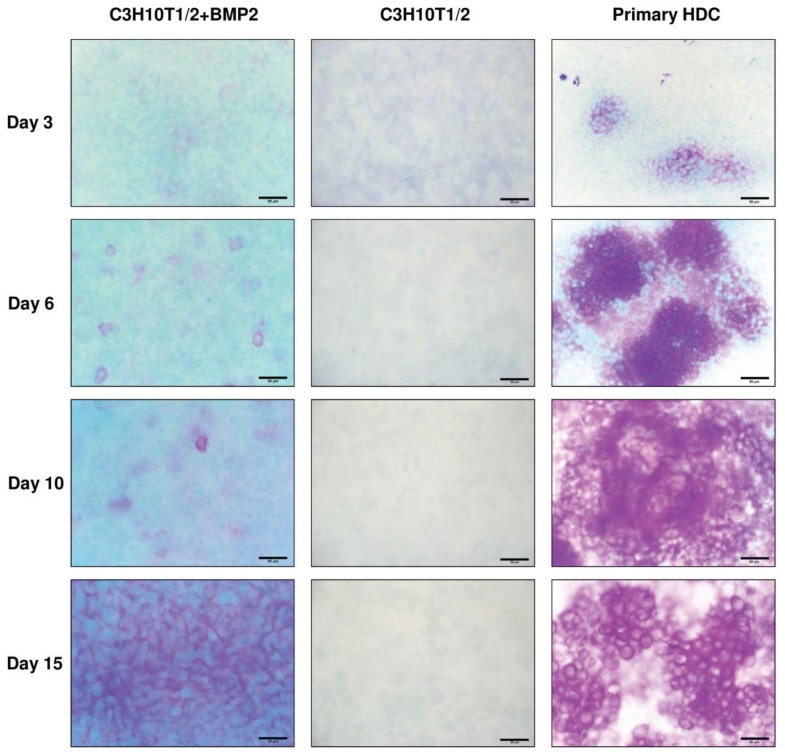
Metachromatic cartilage matrix in micromass cultures on different days of culturing. Metachromatic cartilage areas in HDC were visualised with 0.1% DMMB dissolved in 3% acetic acid (pH 1.0). Original magnification was 40× for all photomicrographs. Scale bar, 50 μm. Metachromatic (purple) areas correspond to cartilage matrix rich in sulphated GAGs. Representative photomicrographs of 3 independent experiments are shown.

Since condensation and precartilaginous nodule formation are the first visible signs of chondrogenesis from the embryonic connective tissue *in vivo* [21], we first looked at whether the two models investigated in this study recapitulated these processes *in vitro*. To this end, routine haematoxylin-eosin (HE) staining procedures were performed on day 3 of culturing to visualise the cellular morphology of the two different micromass cultures. As seen in Figure 1, the cell line-based colony exhibited a substantially different morphology than the primary model. Cells in the limb bud-derived HDC formed numerous nodules with multiple cell layers (marked by arrows), while cellular density remained low in the internodular areas. The fact that differentiation of chondroprogenitor cells into chondroblasts primarily occurs within these foci was confirmed by staining with DMMB: on day 3, metachromatic cartilage matrix could only be detected within the aggregates and no metachromasia was visible in the internodular areas (see Figure 2). By contrast, the central region of the C3H10T1/2-based culture was densely populated with complete lack of foci and internodular areas (Figure 1). Furthermore, cellular behaviour in terms of migratory characteristics was also different in the two models: although some cells have also migrated to the periphery of the primary HD culture, this area remained relatively sparse compared to the cell line-based HDC in which a very high number of cells have spread from the centre and they even formed dense, multiple cellular layers on the periphery.

### 2.2. Micromass Cultures Established from either BMP-2 Overexpressing C3H10T1/2 Cells or Primary Embryonic Limb Bud-Derived Cells Undergo Chondrogenic Differentiation

To assess the amount of metachromatic cartilage matrix accumulation in micromass colonies at select days of culturing, acidic DMMB staining was used. This is a generally accepted approach to verify cartilage matrix formation, as the molecules that are responsible for this phenomenon (*i.e.*, proteoglycans containing highly sulphated glycosaminoglycans) cannot be found at this large abundance in other tissue types. As seen in Figure 2, there is an increasing tendency in the amount of metachromatic ECM areas as differentiation proceeds. However, the temporal pattern of the appearance of relatively large metachromatic territories differs between the examined models. While we observed extensive metachromatic areas within cartilaginous nodules (but not in the internodular areas) in 3-day-old primary HDC, mainly orthochromatic staining was visible in the cell line based models on the same culturing day. By days 6 and 10 of culturing the disparity became even more pronounced between primary HDC and the C3H10T1/2 models. Nonetheless, the BMP-2 overexpressing cell line-based cultures presented higher amounts of metachromatic ECM areas in comparison with the control ones. After 15 days of culturing, the ECM was exclusively metachromatic and enlarged, presumably hypertrophic chondrocytes were also visible in the primary HDC. The size of metachromatic matrix areas also increased in the BMP-2 overexpressing C3H10T1/2 colonies, but it failed to reach the amount detected for primary cultures of the same age. In contrast, no metachromatic territories could be identified in micromass cultures of control C3H10T1/2 cells even on day 15 of culturing. It is of note that the appearance of metachromatic territories in the embryonic limb bud-derived HDC and that in cultures established from the BMP-2 overexpressing variant of C3H10T1/2 was different after staining with DMMB; while the former model exhibited distinct, heavily metachromatic regions that corresponded to cartilaginous nodules, a weaker but relatively homogenous metachromasia was observed in the latter one. Furthermore, considerable orthochromatic territories were also visible throughout the culturing period in colonies of the BMP-2 overexpressing C3H10T1/2.

**Figure 3:**
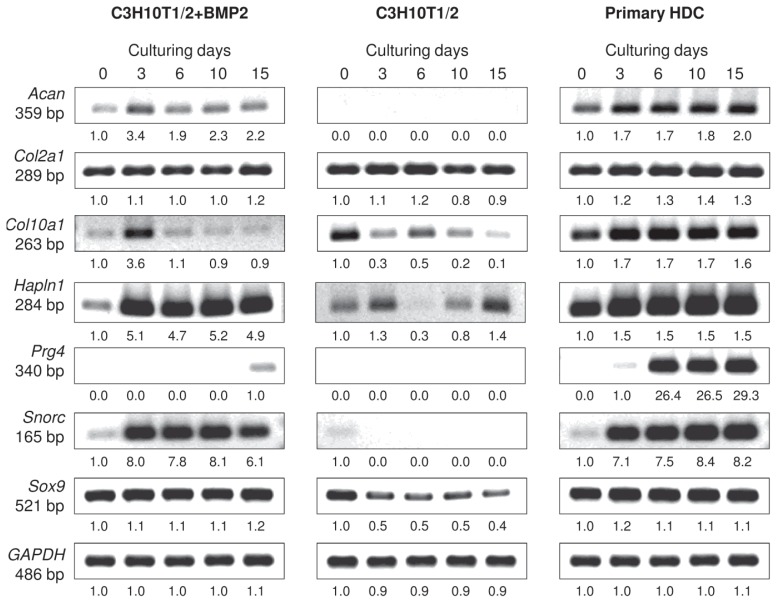
mRNA expression of chondrogenic marker genes in cells of micromass cultures on various days of culturing. *Sox9* codes for the main chondrogenic transcription factor; *Acan* and *Col2a1* are cartilage-specific ECM components; *Hapln1* codes for the hyaluronan and proteoglycan link protein; *Snorc* is a novel cartilage-specific membrane proteoglycan in chondrocytes; *Prg4* is specifically expressed by chondrocytes in the superficial zone of articular cartilage; *Col10a1* is a marker gene for hypertrophic chondrocytes. GAPDH was used as a control. Numbers below gel images represent integrated densities of signals determined using ImageJ 1.46; data were normalized to the value detectable on the earliest day of culturing, day 0 (1.0) where applicable. Representative data of 3 independent experiments. Graphs representing mean values of relative optical densities of PCR results are shown in the Figure S1.

The mRNA expression profiles of specific ECM molecule genes that are associated with differentiation towards the chondrogenic lineage were also examined over the 15-day-long culturing period (Figures 3 and S1). *Sox9*, which encodes a transcription factor responsible for the expression of genes involved in cartilage matrix secretion and is therefore considered as the master gene of chondrogenesis, was found to be expressed at a constant level in both the primary and the BMP-2 overexpressing C3H10T1/2 cultures from the beginning of their HD culturing. Interestingly, mRNA transcripts for *Sox9* were also detected in control C3H10T1/2 micromass cultures, but they gradually decreased by culturing day 15. While *Col2a1*, the gene that codes for the alpha-1 chain of type II collagen, exhibited a constant level of expression in all three micromass cultures (including control C3H10T1/2 colonies that did not produce abundant cartilage ECM), *Acan*, which encodes the core protein of aggrecan, one of the main components of cartilage-specific ECM, only showed strong levels of expression in the embryonic limb bud-derived micromass cultures, and remained at a relatively lower level in the BMP-2 overexpressing C3H10T1/2 colonies. mRNA expression of the gene *Hapln1* coding for the hyaluronan and proteoglycan link protein that connects proteoglycan core proteins to hyaluronan scaffolds was strong in the primary and in the BMP-2 overexpressing model, but only weak signals were detected in the control C3H10T1/2 cultures. The mRNA expression of *Snorc*, a cartilage-specific small transmembrane proteoglycan in differentiating and articular chondrocytes, was confirmed in both the primary and in the BMP-2 overexpressing C3H10T1/2 models commencing from day 3, when cartilage matrix producing chondroblasts have formed. Finally, in the case of the secreted protein lubricin *(Prg4)*, which is specifically expressed by chondrocytes in the superficial zone of articular cartilage and by synoviocytes of the joint capsule, strong signals were only detectable in the embryonic limb bud-derived HDC and only from day 6 of culturing. This observation suggests that the molecular composition of cartilage produced in this model better mimics articular cartilage compared to that in colonies established from the C3H10T1/2 cell line. *Col10a1*, the marker gene that codes for the alpha-1 chain of type X collagen expressed by hypertrophic chondrocytes, showed an almost constant level of expression throughout the entire culturing period in the primary cultures, while signals without any specific temporal pattern could be detected in the C3H10T1/2-based colonies.

### 2.3. Matrix Calcification and Osteogenic Differentiation of C3H10T1/2 Cell Line or Primary Embryonic Limb Bud-Derived Micromass Cultures

**Figure 4:**
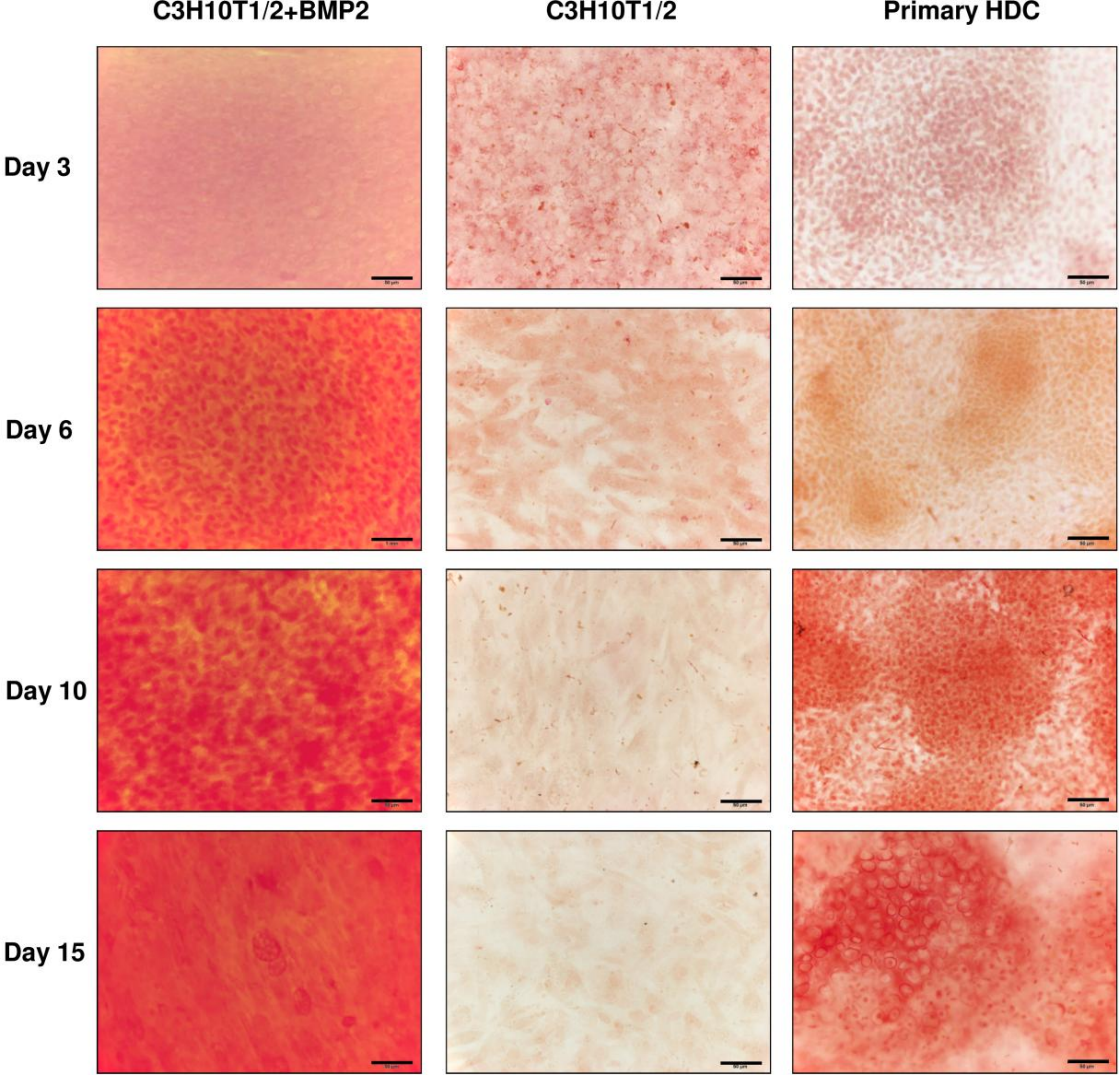
Visualisation of calcified ECM production in HDC on different culturing days. Matrix calcification was detected with Alizarin Red staining. Original magnification was 40× for all photomicrographs. Scale bar, 50 μm. Orange-red areas correspond to extracellular matrix rich in calcium deposits. Representative photomicrographs of 3 independent experiments are shown.

Alizarin Red staining procedure was applied on various days of culturing to assess calcified matrix accumulation in both embryonic limb bud-derived and C3H10T1/2-based micromass cultures. While no specific staining could be observed in the control C3H10T1/2 colonies, a very strong positivity was detected in HDC from the BMP-2 overexpressing C3H10T1/2 cells even from day 6 (Figure 4). By contrast, we could only detect stronger staining for calcified matrix from day 10 in the primary micromass cultures primarily within the cartilaginous nodules. These results suggest that both micromass cultures undergo matrix calcification and presumably hypertrophic transformation of chondrocytes, which precedes endochondral ossification.

**Figure 5:**
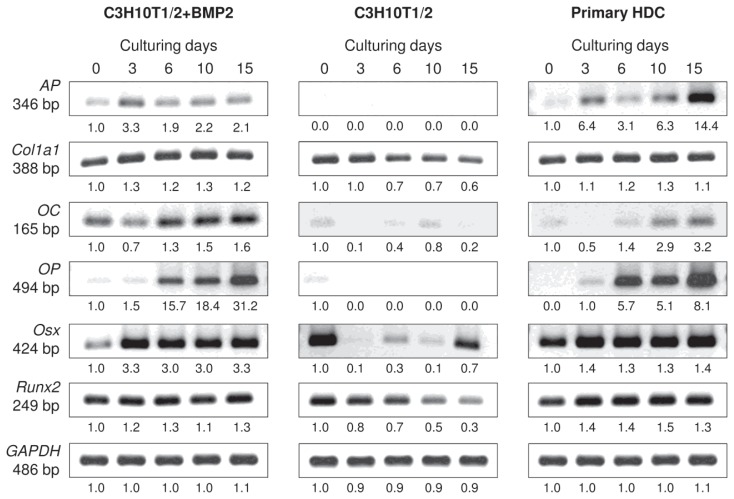
mRNA expression patterns of osteogenic marker genes in cells of micromass cultures on various days of culturing. *Runx2* and *Osx* code for major osteogenic transcription factors; *Col1a1* encodes the alpha-1 chain of type I collagen; *osteocalcin* (*OC*) and *osteopontin* (*OP*) are markers of late stages of osteogenesis; *alkaline phosphatase* (*AP*) is a marker for osteoblast activity. GAPDH was used as a control. Numbers below gel images represent integrated densities of signals determined using ImageJ 1.46; data were normalized to the value detectable on the earliest day of culturing, day 0 (1.0) where applicable. Data is representative of 3 independent experiments. Graphs representing mean values of relative optical densities of PCR results are shown in the Figure S2.

To this end, differentiation towards the osteogenic lineage was also monitored using conventional RT-PCR analysis of lineage-specific genes. The *Runt-related transcription factor-2* (*Runx2*; also known as *Cbfa-1*), the key transcription factor associated with osteoblast differentiation, exhibited a constant expression in the two differentiating micromass cultures but gradually decreased in the control C3H10T1/2 colonies. In contrast, mRNA transcripts for *Osterix* (*Osx*), another important osteoblast-specific transcription factor downstream of *Runx2* and essentially required for bone formation and osteoblast differentiation, were only found to be expressed in a constant manner in the primary and the BMP-2 overexpressing C3H10T1/2 colonies with stronger signals from culturing day 3 (Figures 5 and S2). Although *Osx* mRNA expression could also be detected in control C3H10T1/2 colonies on certain days, it followed a rather irregular pattern. The mRNA expression of *Col1a1* (that encodes the alpha-1 chain of type I collagen) followed a very similar pattern to what has been observed for *Runx2*: a steady but strong expression was seen in the BMP-2 induced C3H10T1/2 and in the primary cultures, whereas a decline was detected in the control C3H10T1/2 colonies. mRNAs for *osteocalcin* (*OC*) and *osteopontin* (*OP*), markers of late stages of osteogenesis, however, exhibited expression sequences similar to those of *Osx*: both markers showed stronger signals towards the end of the 15-day-long culturing period and could mainly be detected in the BMP-2 overexpressing C3H10T1/2 and in the limb bud-derived micromass cultures. *Alkaline phosphatase* (*AP*), another important marker for osteoblast activity, was also only expressed in the BMP-induced and the primary micromass cultures, with a very strong upregulation by day 15 in the latter model.

### 2.4. Differentiation towards the Adipogenic Lineage Is also Characteristic of the Micromass Models Studied

**Figure 6:**
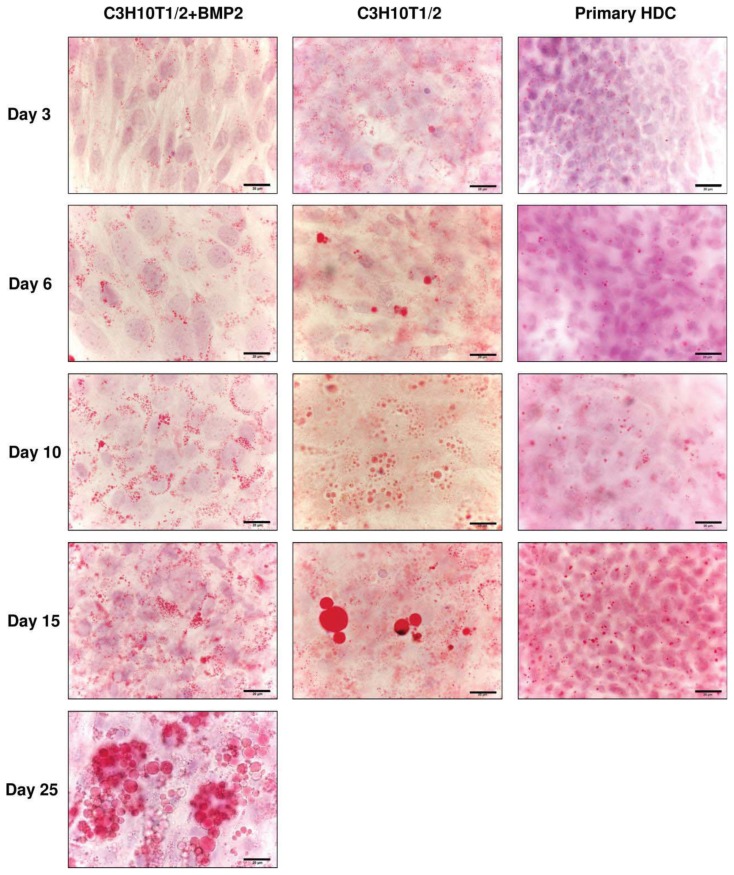
Analysis of adipogenesis in micromass cultures established from C3H10T1/2 cell line (control and BMP-2 overexpressing variants) and embryonic limb bud-derived mesenchymal cells on select days of culturing. Oil Red O was applied to selectively stain cytoplasmic lipid droplets (red). Nuclei were counterstained with haematoxylin (blue). Original magnification was 100× for all photomicrographs. Scale bar, 20 μm. Representative photomicrographs of 3 independent experiments are shown.

As C3H10T1/2 is a multipotent mesenchymal cell line, pathways of differentiation other than chondro- and osteogenesis are necessary to consider. To identify whether cells in the two different models applied in this study underwent differentiation towards the adipogenic lineage and acquired an adipocyte-like phenotype, lipid droplet-specific Oil Red O staining procedures were performed on select days of culturing. Haematoxylin nuclear staining was also applied on cultures to facilitate identification of cells. As seen in Figure 6, cells in the embryonic limb bud-derived micromass cultures did not exhibit large lipid droplets even at later stages (day 15); only small lipid droplets that are structural components of chondrocytes were visible [22]. In contrast to complete lack of adipocyte-like cells in primary HDC, C3H10T1/2-based cultures (both the control and the BMP-2 overexpressing variant) showed cells with strong Oil Red O-positive lipid droplets especially at later time points (days 10 and 15). Interestingly, many adipocyte-like cells could be identified even in the control C3H10T1/2-based micromass cultures (Figure 6). The number of large cells with multiple lipid droplets that can be considered as precursors of white adipocytes further increased at later stages: by day 25 of culturing, these cells virtually outnumbered other cell types at the periphery and in the superficial layer over the centre of the cell line based micromass culture. This phenomenon could not be observed in the embryonic limb bud-derived HDC (data not shown).

**Figure 7:**
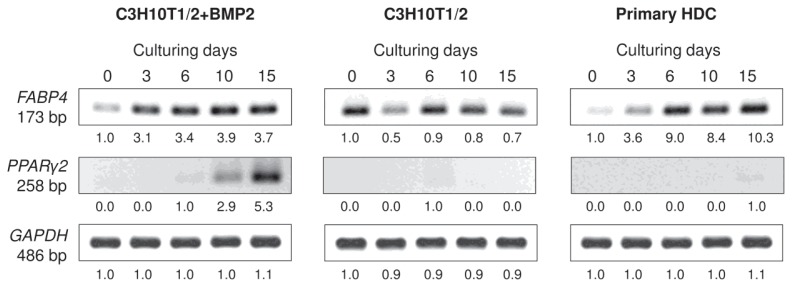
Expression patterns of mRNAs for adipogenic marker genes in cells of micromass cultures on various days of culturing. *FABP4* is an adipocyte-specific marker; *PPAR*γ*2* codes for an adipocyte-specific nuclear hormone receptor and key regulator of adipocyte differentiation. GAPDH was used as a control. Numbers below gel images represent integrated densities of signals determined using ImageJ 1.46; data were normalized to the value detectable on the earliest day of culturing, day 0 (1.0) where applicable. Data is representative of 3 independent experiments are shown Graphs representing mean values of relative optical densities of PCR results are shown in the Figure S3.

The mRNA expression patterns of genes that mark differentiation towards the adipogenic lineage were analysed by RT-PCR. mRNA transcripts of the adipocyte-specific fatty acid binding protein-4 *FABP4* (adipocyte protein-2) could be detected in all three micromass cultures with a strong upregulation in both the BMP-2 overexpressing variant of the C3H10T1/2 cultures and in the primary HDC at later stages of culturing (Figures 7 and S3). By contrast, stronger signals of the peroxisome proliferator-activated receptor gamma 2 *(PPARγ2)*, an adipocyte-specific nuclear hormone receptor and key regulator of adipocyte differentiation, could only be detected in the BMP-2 overexpressing C3H10T1/2 derived HDC and only at later developmental stages, which further supports our observations based on Oil Red O staining procedures.

### 2.5. mRNA Transcripts of Pluripotency Factors Are Detectable Even at Later Stages in Micromass Cultures

**Figure 8:**
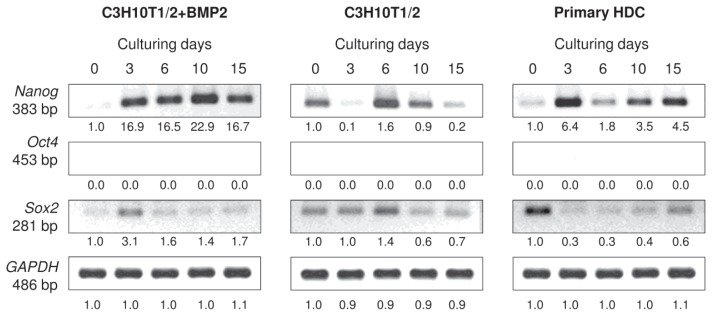
Effect of micromass culturing conditions on mRNA expression of pluripotency markers. *Nanog*, *Sox2* and *Oct-4* are key genes that are essential to maintain pluripotency and self-renewal of ESCs. GAPDH was used as a control. Numbers below gel images represent integrated densities of signals determined using ImageJ 1.46; data were normalized to the value detected on day 0 (1.0). Representative data of 3 independent experiments. Graphs representing mean values of relative optical densities of PCR results are shown in the Figure S4.

Another aspect we were interested in is whether some of the cells maintained a pluripotent state in HDC. Therefore, mRNA expression patterns of key genes (*Nanog*, *Sox2* and *Oct-4*) expressed in embryonic stem cells (ESCs) were analysed in all three micromass cultures (Figures 8 and S4). While no mRNA transcripts for *Oct-4*, a homeodomain transcription factor that is essential to maintain self-renewal of ECSs, were detectable in any of the models, *Nanog* and *Sox2* were identifiable in all cultures, although with varied expression patterns. *Nanog* exhibited a strong upregulation in the BMP-2 overexpressing cultures and also in the primary HDC from day 3, whereas *Sox2* showed a relatively stronger expression at the beginning of culturing for both models and became downregulated as differentiation progressed with slight elevation in 15-day-old primary HDC. No clear pattern could be established for these two markers in the unstimulated C3H10T1/2 cultures.

## 3. Discussion

This study provides a detailed comparative analysis of osteo-, chondro- and adipogenic differentiation potential of widely applied murine micromass models, *viz*. a primary, embryonic limb bud-derived system *vs.* a multipotent mesenchymal cell line-based one. For the latter model used in particular study, C3H10T1/2 cells contain a plasmid that maintains a constant BMP-2 overexpression and secretion into the culture medium that is independent of external sources [20]. These model systems have been extensively used to study various steps of chondrogenesis (both early and late stages) [2,19,20,23,24]; however, a comparative study to elucidate the extent and quality of differentiation potential into various cell types of mesenchymal origin has not been performed. Although mRNA expression profiles of several chondrogenic and osteogenic marker genes have been explored in both models, a direct comparison of a wide array of genes under identical culture conditions and sampling intervals was lacking.

A number of signalling pathways were demonstrated to play essential roles in the complex process of cartilage formation prior to endochondral ossification at various developmental stages. These include the Notch and Wnt signalling pathways during mesenchymal condensation, and utilise several secreted factors (morphogens) such as TGF-β, BMP, insulin-like growth factor (IGF), and fibroblast growth factor (FGF) to modulate proliferation and differentiation of chondroprogenitor cells, chondrocyte maturation and hypertrophy [25]. The BMP pathway has been indicated to enhance *in vitro* differentiation of mesenchymal cells towards various lineages (mainly bone and cartilage) [26]. Among BMPs, BMP-2 is specifically expressed in the condensing mesenchyme of the embryonic limb and vertebrae and is believed to play a critical role in the patterning and formation of the skeleton [27]. Depending on a set of factors and culturing conditions, BMP-2 in particular can induce differentiation of C3H10T1/2 and 3T3 cells towards different mesenchymal lineages, such as adipocytes, osteoblasts, or chondrocytes [28].

In our study, one of the most obvious differences between the embryonic limb bud-derived and the C3H10T1/2 cell line-based micromass cultures was the presence or absence of distinct prechondrogenic nodules. It is probably the high number of intercellular connections (primarily *N*-cadherins) between differentiating chondroprogenitor cells in primary micromass cultures [1] that creates a more tissue-like environment, which may resemble the one that characterises *in vivo* chondrogenesis. However, these same types of intercellular connections have also been shown in C3H10T1/2-derived micromass cultures [29]. Moreover, cells of the three types of cultures were also different from each other in terms of migratory potentials: while the embryonic limb bud-derived cells stayed together in the initial spot cultures, the control C3H10T1/2 cells migrated so intensely that the initial spot culture was not identifiable after a few days of culturing. The BMP-2 overexpressing C3H10T1/2 cells were also motile, although only cells at the periphery of the spot cultures were characterised by higher migratory potentials and cellular density remained high at the centre. This observation is supported by the results of Haas and Tuan according to which induction by BMP-2 is required for the upregulation of *N*-cadherin expression [29]. The observed difference in migratory features can also account for the varied morphology of micromass cultures.

Since the primary aim of this study was to analyse the extent and quality of chondrogenic differentiation, we first compared the mRNA expression profiles of chondrocyte-specific marker genes. Both the BMP-2 overexpressing C3H10T1/2 cell line-based and the primary micromass models showed a constant mRNA expression profile for most of the basic chondrocyte-specific genes (*i.e.*, *Sox9*, *Acan*, *Col2a1*, and *Hapln1*); furthermore *Snorc*, a novel chondrocyte-specific transmembrane chondroitin sulphate proteoglycan [30] exhibited strong mRNA expression in both models. However, *Prg4* that encodes the glycoprotein lubricin (superficial zone protein) and is a hallmark of chondrocytes in the superficial layer of articular cartilage [31], was only found to be heavily expressed in embryonic limb bud-derived primary micromass cultures, suggesting that the cartilage matrix produced in these cultures more closely resembles that of native cartilage of limb primordia. This was also confirmed by analysis of metachromatic cartilage ECM morphology, distribution and quality. Moreover *Col10a1*, which is primarily expressed by hypertrophic chondrocytes, was observed throughout the entire culturing period in the primary cultures *vs.* a relatively weaker expression pattern in the cell line-based model. It is of note, however, that collagen type X is reported to be a natural structural supporting component of mouse articular cartilage throughout development and growth [32].

Since in general, type X collagen is considered as a marker of chondrocyte maturation and hypertrophy during endochondral ossification [33], we also performed a more detailed comparative analysis of osteogenic marker genes in the two models investigated. Interestingly, mRNA transcripts of the major osteogenic transcription factors (*Runx2* and *Osx*) that are both necessary for osteoblast differentiation [34] were readily identifiable in the transcriptome of both differentiating models even on day 0 of culturing (whilst being rapidly downregulated in the unstimulated control C3H10T1/2 cultures), suggesting that differentiation towards the osteogenic lineage was present even at the beginning of HD culturing. The presence of osteoprogenitor cells has been confirmed by earlier studies in both primary micromass cultures [35] and in BMP-2 induced C3H10T1/2-based HDC [36]. The observation that osteogenesis commenced at relatively early time points in these cultures was further supported by the fact that mRNA expressions of some markers (*Col1a1* and *AP*) could also be readily detected even at the beginning of culturing, with a very strong upregulation of the latter gene in the primary HDC, probably owing to increased *Osx* activity. Upregulation of *AP* transcripts towards later stages of culturing was also observed by another group using the same model [24]. Nonetheless, late osteogenic markers (*OC* and *OP*) generally only showed stronger signals towards the end of the 15-day-long culturing period. For *OC*, these data are also in a good correlation with results gained earlier using primary micromass cultures [24,35] and in BMP-2 and BMP-7 induced C3H10T1/2 cultures [37]. Interestingly, however, mRNA transcript levels in C3H10T1/2 cultures differentially responded to treatments with various BMPs; BMP-7 resulted in a biphasic mRNA expression, whereas BMP-2 caused *OP* upregulation only at later time points [37], which coincides well with our own observations. At the same time, *OP* mRNA expression patterns have not been investigated earlier in primary murine embryonic limb bud-derived HDC. Nevertheless, our results regarding *OC* and *OP* transcript expression in the BMP-2 overexpressing C3H10T1/2 micromass model are in a perfect correlation with the findings of Ahrens and colleagues using the same model system [20].

The above results indicate that cells in both the primary and the BMP-2 overexpressing C3H10T1/2 model system express mRNA transcripts for the same osteogenic markers at almost identical temporal patterns. This, taken together with data drawn from visualising ECM calcification with Alizarin Red staining, implies that both model systems recapitulate embryonic endochondral bone formation at approximately the same pace and *via* the same pathway. Noteworthy, however, that the expression of markers that are hallmarks of articular cartilage (*i.e.*, *Snorc* and *lubricin*) coincided with bone markers (e.g., *OC* and *OP*), which suggests that chondro- and osteogenesis occurred simultaneously, rather than sequentially, in these models. Nevertheless, interpretation of data concerning the mRNA expression of these markers should be performed with caution as *Runx2*, *Osx* and even *OC* have also been reported to be amongst the main regulators of late chondrocyte differentiation and are also expressed in prehypertrophic and hypertrophic chondrocytes [35]. Furthermore, the fact that the cells expressing mRNA transcripts for *OC* were mainly located in the internodular areas of primary limb bud-derived micromass cultures and were thus functionally distinct from differentiating chondrocytes within cartilaginous aggregates should also be taken into account [35].

Since C3H10T1/2 cells are reported to differentiate towards the adipogenic lineage and as BMPs are also known to induce adipogenic differentiation [20], we also looked at whether cells in the models investigated in this study showed signs of adipogenesis. For this, we examined the expression of mRNA transcripts of the adipocyte-specific factors *FABP4* and *PPARγ2*. Fatty acid binding proteins (FABP) are lipid transporters expressed in multiple tissues; however, *FABP4* (also known as adipocyte protein-2) is known to be specific to adipocytes and is also a marker for adipocyte precursors [38]. We found that *FABP4* could be detected in all three micromass cultures with a strong upregulation in both the BMP-2 overexpressing C3H10T1/2-based micromass cultures and in the primary HDC at later stages of culturing. This finding shows a good correlation with results gained with Oil Red O staining protocols that confirmed the presence of lipid droplet-containing adipocytes in both C3H10T1/2-derived HDC models. In contrast, transcripts for *PPARγ2*, which is a key regulator of adipocyte differentiation [39], could only be detected in the BMP-2 overexpressing C3H10T1/2-derived HDC and only at later developmental stages, suggesting that adipogenesis should only be considered in these cultures. Indeed, on day 25 of culturing, a massive number of single, large lipid droplet-containing cells (probably adipocytes) were observed both at the periphery and in the superficial layer over the centre of micromass cultures. It is of note that accumulation of lipid droplets could also be detected in cells of the unstimulated C3H10T1/2 cultures. In cells of primary micromass cultures, cells with small and sparse lipid droplets were observable; these are probably mature chondrocytes that contain lipid droplets under physiological circumstances [22].

Apart from the well-established differentiated phenotypes, we also aimed to determine whether certain cells in micromass cultures maintained an undifferentiated (pluripotent) state. To this end, mRNA transcripts of the hallmarks of pluripotency (*Nanog*, *Sox2* and *Oct-4*) were analysed in all three micromass cultures. *Nanog* is a homeodomain transcription factor; Sox2 (SRY2) is a high mobility group box-containing transcription factor; and *Oct*-*4* (POU5F1) is a homeodomain transcription factor of the POU family; they are all critically involved in self-renewal of undifferentiated ESCs [40]. Of these genes, *Nanog* and *Sox2* were identifiable in all models, although with opposite expression patterns: *Nanog* exhibited a strong upregulation from day 3, whereas *Sox2* showed a relatively stronger expression at the beginning of culturing and became downregulated as differentiation progressed. Interestingly, no mRNA transcripts for *Oct-4* were detected in these models. These findings suggest that some cells remain undifferentiated in the models investigated and not all cells undergo terminal differentiation towards any of the lineages discussed above.

## 4. Experimental Section

### 4.1. Cell Culturing

#### 4.1.1. Micromass Cultures Established from C3H10T1/2 Cells

The murine mesenchymal cell line C3H10T1/2 was purchased from the American Type Culture Collection (ATCC; Rockville, MD, USA). The BMP-2 overexpressing C3H10T1/2 cell line, which was permanently transfected with cDNA encoding the human bone morphogenic protein BMP-2 cloned into the eukaryotic expression vector pMBC-2T-fl, was established in the laboratory of G. Gross and was a kind gift from that research group. Constitutive transcription of the construct was achieved by the long terminal repeat (LTR) promoter of the myeloproliferative sarcoma virus (MPSV) and terminated by a poly(A) site from SV40; selection of clones was performed by cotransfection with pBSpACΔp plasmid that confers resistance against puromycin [20]. Monolayer cultures of the BMP-2 overexpressing and control C3H10T1/2 cells were routinely maintained in standard 75 cm^2^ cell culture flasks, or to obtain high cell numbers, in 150 cm^2^ polystyrene tissue culture dishes (Orange Scientifique, Braine-l’Alleud, Belgium). Cells were cultured in high glucose (4.5 g·L^−1^) Dulbecco’s modified Eagle’s medium (DMEM; Sigma-Aldrich, St. Louis, MO, USA) supplemented with 10% (*v*/*v*) foetal calf serum (FCS; Gibco, Gaithersburg, MD, USA), 0.5 mM alanyl-glutamine (Ala-Gln) as a substitute for l-glutamine (Sigma-Aldrich), 6.6 μg·mL^−1^ ampicillin and 100 μg·mL^−1^ streptomycin (TEVA, Debrecen, Hungary). The culture medium of the BMP-2 overexpressing cells contained additional 5 μg·mL^−1^ puromycin (Sigma-Aldrich). Cultures were incubated in a humidified CO_2_ incubator at 37 °C. Cells were passaged with 0.25% trypsin-EDTA (Sigma-Aldrich) dissolved in phosphate buffered saline (PBS) for 2 min at 37 °C when they reached ~80% confluency.

To establish micromass cell cultures from BMP-2 overexpressing and control C3H10T1/2, cells were harvested by brief centrifugation (at 800 × *g* for 10 min) following trypsinisation. The number of cells was determined using a haemocytometer, and cellular density was set to 1.5 × 10^7^ cells·mL^−1^ in DMEM supplemented with 10% FCS. 30 or 100 μL droplets of the cell suspension were inoculated into 35 mm plastic tissue culture dishes (Orange Scientifique). The cells were allowed to attach to the surface at 37 °C and 5% CO_2_ for 2 h, and then the dishes were flooded with the culture medium, which was changed on every second day. Day of inoculation was considered as day 0 of culturing. Micromass cultures were maintained for up to 15 days.

#### 4.1.2. Primary Embryonic Mesenchymal Micromass Cultures

Mouse embryonic limb bud-derived mesenchymal cell cultures were established according to the standard protocol used by our laboratory on chicken high density cultures [41] with minor modifications for mice. NMRI laboratory mice were mated overnight and mating was confirmed by the presence of a vaginal plug (considered as day 0 of gestation). On day 11.5 of gestation, pregnant female mice were sacrificed by cervical dislocation, according to the regulations defined in the licence obtained from the University of Debrecen Committee of Animal Research (11/2010/DE MÁB). For each animal, the uterus was removed and washed in sterile calcium and magnesium-free PBS (CMF-PBS), pre-heated to 37 °C. E11.5 embryos were then isolated from the uterus, pooled and washed several times in CMF-PBS.

To establish primary micromass cultures, distal parts of fore and hind limb buds of embryos were removed under a dissecting microscope using two pairs of sharp forceps and pooled in CMF-PBS. Once all limb buds have been collected, they were transferred into 0.25% trypsin-EDTA (Sigma-Aldrich) and incubated at 37 °C in a CO_2_ incubator (5% CO_2_ and 80% humidity) for 1 h. The enzymatic digestion was terminated by the addition of equal volume of FCS (Gibco). Limb buds were disaggregated by gentle aspiration using 5 mL plastic pipette tips until no clumps remained and cells were filtered through a 20-μm pore size plastic filter unit (Millipore, Billerica, MA, USA) to yield a single cell suspension of mesenchymal cells. After a brief centrifugation (at 800× *g* for 10 min), cell pellet was resuspended in Ham’s F12 medium (Sigma-Aldrich) supplemented with 10% FCS at a concentration of 1.5 × 10^7^ cells mL^−1^ and 30 or 100 μL droplets were inoculated into 35 mm plastic tissue culture dishes (Orange Scientifique). After allowing the cells to attach to the surface for 2 h at 37 °C in a CO_2_ incubator, 2 mL of Ham’s F12 culture medium supplemented with 10% FCS, 0.5 mM alanyl-glutamine (Ala-Gln) as a substitute for l-glutamine (Sigma-Aldrich), and antibiotics/antimicotics (penicillin, 50 U·mL^−1^; streptomycin, 50 μg·mL^−1^; fungizone, 1.25 μg·mL^−1^; TEVA, Debrecen, Hungary) was added. Day of inoculation was considered as day 0 of culturing. High density cultures were maintained at 37 °C in a CO_2_ incubator for 15 days. The medium was changed on every second day.

### 4.2. mRNA Expression Analysis Using Reverse Transcription Followed by PCR

On various days of culturing (*i.e.*, on days 0, 3, 6, 10 and 15), micromass cultures established from 100 μL droplets of either C3H10T1/2 or limb bud-derived mesenchymal cells were washed three times with RNase-free physiological NaCl, then the cultures were snap-frozen in liquid nitrogen and stored at −70 °C. Cell cultures were dissolved in TRIzol (Applied Biosystems, Foster City, CA, USA), and following addition of 20% RNase-free chloroform (Sigma-Aldrich) samples were centrifuged at 10,000× *g* for 15 min at 4 °C. Total RNA-containing samples were incubated in 500 μL RNase-free isopropanol at −20 °C for 1 h, total RNA was dissolved in nuclease-free water (Promega, Madison, WI, USA) and stored at −70 °C.

The composition of the assay mixture (20 μL) for reverse transcriptase (RT) reactions was as follows: 1000 ng total RNA; 0.25 μL RNase inhibitor; 2 μL random primers; 0.8 μL dNTP Mix (4 mM); 50 units (1 μL) of MultiScribe™ RT in 1× RT buffer (High Capacity RT kit; Applied Biosystems, Foster City, CA, USA). cDNA was transcribed at 37 °C for 2 h.

Amplifications of specific cDNA sequences were carried out using specific primer pairs that were designed by Primer Premier 5.0 software (Premier Biosoft, Palo Alto, CA, USA) based on murine nucleotide sequences published in GenBank and purchased from Integrated DNA Technologies, Inc. (IDT; Coralville, IA, USA). The specificity of custom-designed primer pairs was confirmed *in silico* by using the Primer-BLAST service of NCBI. Nucleotide sequences of forward and reverse primers and reaction conditions are shown in Table 1. PCR reactions were carried out in a final volume of 25 μL containing 1 μL forward and 1 μL reverse primers (10 μM), 0.5 μL cDNA, 0.5 μL dNTP Mix (200 μM), and 1 unit (0.2 μL) of GoTaq^®^ DNA polymerase in 1× Green GoTaq^®^ Reaction Buffer (Promega) in a programmable thermal cycler (Labnet MultiGene™ 96-well Gradient Thermal Cycler; Labnet International, Edison, NJ, USA) with the following protocol: 2 min at 95 °C for initial denaturation followed by 35 repeated cycles of denaturation at 94 °C for 30 s, primer annealing for 45 s at an optimised temperature for each primer pair (see Table 1), and extension at 72 °C for 90 s. After the final cycle, further extension was allowed to proceed for another 7 min at 72 °C. PCR products were analysed using horizontal gel electrophoresis in 1.2% agarose gels containing ethidium bromide at 90 V constant voltage. Optical density of PCR product signals was determined by using ImageJ freeware (Image Processing and Analysis in Java) version 1.46 [42].

### 4.3. Histological Analysis of Micromass Cultures

#### 4.3.1. Investigation of Cellular Morphology of Micromass Cultures by Conventional Haematoxylin and Eosin Staining

Micromass cell cultures established from 30 μL droplets of the cell suspensions were cultured on the surface of round 30 mm coverglasses (Menzel-Gläser, Menzel GmbH, Braunschweig, Germany) placed into 35 mm plastic culture dishes. On day 3 of culturing, after washing with PBS, cultures were fixed with a 4:1 mixture of absolute ethanol and 40% formaldehyde. After rehydration in a descending series of ethanol cells were stained with Gill’s haematoxylin No. 2 and eosin Y (1% aqueous solution; Bio Optica Milano S.p.A., Italy). Briefly, cultures were first immersed in haematoxylin for 20 s, rinsed in running tap water for 5 min, and after washing in distilled water, eosin was applied for 2 min. Eosin was washed and cultures were dehydrated in ascending series of alcohol, and after a final wash in xylene, colonies were mounted onto glass slides using DPX mounting medium (Sigma-Aldrich). Photomicrographs of the cultures were taken using an Olympus DP72 camera on a Nikon Eclipse E800 microscope (Nikon Corporation, Tokyo, Japan). Images were acquired using cellSense Entry 1.5 software (Olympus, Shinjuku, Tokyo, Japan).

#### 4.3.2. Assessment of Chondrogenic Differentiation by Low-pH Metachromatic Staining with Dimethyl-Methylene Blue

On various days of culturing (see above), cultures seeded from 30 μL droplets of the cell suspensions cultured on the surface of round coverglasses were rinsed with PBS and fixed in a 4:1 mixture of absolute ethanol and 40% formaldehyde and following rehydration in a descending series of ethanol cells were stained with 0.1% (*w*/*v*) 1,9-dimethyl-methylene blue (DMMB, Sigma-Aldrich) dissolved in 3% acetic acid (pH 1.0). Surplus dye was washed in acetic acid and cultures were mounted in gum arabic. Photomicrographs of the cultures were taken using an Olympus DP72 camera on a Nikon Eclipse E800 microscope.

#### 4.3.3. Assessment of Matrix Mineralisation by Alizarin Red S Staining

To demonstrate the extent of matrix calcification, micromass cultures established from 30 μL droplets of the cell suspensions onto 30 mm round coverglasses were used. Cultures were fixed with the same fixative as above on various days of culturing, and then stained with 2% (*w*/*v*) Alizarin Red S (Sigma-Aldrich) dissolved in distilled water (pH 4.2; adjusted with 10% ammonium hydroxide) for 2 min. Excess dye was removed with aspiration, coverslips were blotted and were dipped into acetone (20 times) for dehydration, and then into acetone-xylene (1:1) mixture (another 20 times). Coverglasses were finally mounted onto glass slides using DPX mounting medium (Sigma-Aldrich). Photomicrographs of the cultures were taken as described above.

#### 4.3.4. Assessment of Lipid Accumulation by Staining with Oil Red O

To assay adipogenic differentiation and lipid accumulation in micromass cultures established from 30 μL of the cell suspensions from either C3H10T1/2 or primary mouse embryonic limb bud-derived cells, Oil Red O staining procedure was performed on respective days of culturing (see above). Cultures were fixed in 10% formalin for 60 min at room temperature. Formalin was washed with distilled water, and cultures were rinsed in 60% isopropanol for 5 min. Isopropanol was removed from cultures with aspiration, followed by addition of 2 mL of Oil Red O working solution (prepared as follows: 0.3% (*w*/*v*) Oil Red O (Sigma-Aldrich) stock solution was made using 99% isopropanol; Oil Red O working solution was prepared by mixing 3 volumes of Oil Red O stock solution with 2 volumes of distilled water) for 5 min at room temperature. After the incubation, the working solution was discarded, surplus dye was removed with tap water, and then Gill’s haematoxylin No. 2 was applied for 20 s to provide nuclear background staining. Haematoxylin was rinsed with tap water, followed by mounting the cultures with gum arabic. Photomicrographs of the cultures stained with Oil Red O and haematoxylin were taken as described above.

## 5. Conclusions

We can conclude that in the two differentiating micromass cultures studied, all three major differentiation pathways (*i.e.*, chondrogenic, osteogenic and adipogenic), along with undifferentiated cells that maintain the mRNA expression of key pluripotency factors, could be confirmed at both mRNA and morphological levels. In this way, commitment and differentiation towards these distinct lineages occur in a parallel fashion, rather than sequentially, in these cultures. Furthermore, mRNAs of the key lineage-specific transcription factors (*i.e.*, *Sox9; Runx2* and *Osx*; and *PPARγ2*) could be readily identified as early as day 0 of culturing; whereas towards day 15, transcripts of marker genes for all three lineages (*i.e.*, *Snorc* and *PRG4* for “good quality” cartilage; *AP*, *OC* and *OP* for bone; and *FABP4* for adipocytes) were found to be upregulated. The heterogeneity of these cultures, therefore, has to be taken into account during data interpretation. Furthermore, it is of note that apart from the lineage-specific transcription factors themselves, neither chondrogenic nor osteogenic marker genes could be detected in the transcriptome of unstimulated C3H10T1/2-derived micromass cultures (the only exception being *Col2a1*), suggesting that the inductive effect of autocrine/paracrine secreted factors (primarily BMP-2) is indispensable for lineage-specific differentiation to take place. Moreover, adipogenesis occured by day 15 even in unstimulated C3H10T1/2 cultures without the administration of any factors, which was further enhanced in the BMP-2 overexpressing C3H10T1/2 model, suggesting that formation of adipocytes might be a default pathway for this cell line at the given cellular density.

In fact, heterogeneity is a general problem also encountered in the field of mesenchymal stem cell research. In particular, the stem cell niche in cartilage is not yet characterised in great detail, albeit progenitor cells with the potential to differentiate into secretory cells have already been described in both healthy and diseased cartilage tissue. Consequently, despite the efforts that have been made, establishing a chondrogenic mesenchymal stem cell line that forms functional hyaline cartilage *in vivo* has still been the Holy Grail in cartilage research [43]. Nevertheless, these data taken together with the morphology of primary micromass cultures and the presence of hypertrophic chondrocytes at later time points gives further evidence in support of the observation that the embryonic limb bud-derived model more closely recapitulates embryonic cartilage formation followed by endochondral bone formation. This is consistent with the observations of other research groups [24], and can therefore still be considered as a valid tool to study *in vitro* chondrogenesis. However, we found that although these models provide a powerful tool to assess the temporal and functional relationship among various signalling pathways and important modulators of mesenchymal differentiation, the results gained by using these specific experimental approaches to assess *in vitro* differentiation of mesenchymal cells towards distinct lineages must be interpreted with considerable caution. Since the transcriptome of micromass cultures does not necessarily reflect the actual proteome, further studies are needed to better characterise differentiation into these lineages at the protein level.

## Supplementary Information



## Figures and Tables

**Table 1:** Nucleotide sequences, amplification sites, GenBank accession numbers, amplicon sizes and PCR reaction conditions for each primer pair are shown.

Gene	Primer	Nucleotide sequence (5′→3′)	GenBank Accession No.	Annealing temperature	Amplicon size (bp)
1. Chondrogenic marker genes					
Sox9	sense	GTA CCC GCA TCT GCA CAA CG (378–397)	NM_011448	62 °C	521
	antisense	GTG GCA AGT ATT GGT CAA ACT CAT T (874–898)			
Aggrecan core protein (Acan)	sense	CGG GAA GGT TGC TAT GGT G (782–800)	NM_007424.2	59 °C	359
	antisense	CCT GTC TGG TTG GCG TGT A (1122–1140)			
Collagen II (Col2A1)	sense	AAA GAC GGT GAG ACG GGA GC (1900–1919)	NM_001113515	63 °C	289
	antisense	GAC CAT CAG TAC CAG GAG TGC C (2167–2188)			
Hapln1	sense	GGC TCA GGA ATC CAC AAA (217–234)	BC066853	55 °C	284
	antisense	GGA AAG TAA GGG AAC ACC A (482–500)			
Lubricin (Prg4)	sense	CGA GGT CAT TAT TTC TGG (64–81)	NM_021400	51 °C	340
	antisense	TCA TTG GCT CCT GTT TAT (386–403)			
Snorc	sense	CCC TGT GGA ACG AGC CTA T (101–119)	NM_028473	58 °C	165
	antisense	CAA GCG ATC CTC CAT CCT G (247–265)			
2. Osteogenic marker genes					
Alkaline phosphatase (ALPL)	sense	GAA GTC CGT GGG CAT CGT (474–491)	NM_007431	59 °C	346
	antisense	CAG TGC GGT TCC AGA CAT AG (801–820)			
Collagen I (Col1A1)	sense	GGG CGA GTG CTG TGC TTT (237–254)	BC050014	62 °C	388
	antisense	GGG ACC CAT TGG ACC TGA A (606–624)			
Collagen X (Col10A1)	sense	TTC TGG GAT GCC GCT TGT C (1602–1620)	NM_009925	61 °C	263
	antisense	TCG TAG GCG TGC CGT TCT T (1846–1864)			
Osteocalcin	sense	AGC AGG AGG GCA ATA AGG (110–127)	NM_007541	57 °C	165
	antisense	CGT AGA TGC GTT TGT AGG C (256–274)			
Osteopontin	sense	GCT GAA GCC TGA CCC ATC T (126–144)	X51834	59 °C	494
	antisense	TCC CGT TGC TGT CCT GAT (602–619)			
Osterix	sense	CCC TTC CCT CAC TCA TTT CC (271–290)	AF184902	59 °C	424
	antisense	CAA CCG CCT TGG GCT TAT (677–694)			
Runx2	sense	GGA CGA GGC AAG AGT TTC A (595–613)	NM_001146038	58 °C	249
	antisense	TGG TGC AGA GTT CAG GGA G (825–843)			
3. Adipogenic marker genes					
Pparg2	sense	TGC CTA TGA GCA CTT CAC (62–79)	AY208183	52 °C	258
	antisense	TGA TCG CAC TTT GGT ATT (302–319)			
FABP4	sense	AAA GAA GTG GGA GTG GGC (64–81)	NM_024406	58 °C	173
	antisense	CTG TCG TCT GCG GTG ATT (219–236)			
4. Pluripotency factors					
Nanog	sense	GCC CTG ATT CTT CTA CCA (194–211)	AY278951	54 °C	383
	antisense	AGA TGC GTT CAC CAG ATA G (558–576)			
OCT4 (Pou5f1)	sense	GCA CGA GTG GAA AGC AAC (286–303)	NM_013633	56 °C	453
	antisense	CGG GCA CTT CAG AAA CAT (721–738)			
Sox2	sense	AAC CAG CGC ATG GAC AGC (466–483)	U31967	63 °C	281
	antisense	TCG GAC TTG ACC ACA GAG CC (727–746)			
5. Control gene					
GAPDH	sense	TGG CAA AGT GGA GAT TGT TG (69–88)	NM_008084	60 °C	486
	antisense	GTC TTC TGG GTG GCA GTG AT (535–554)			

## Abbreviations

AP: alkaline phosphatase
BMP: bone morphogenic protein
CMF-PBS: calcium and magnesium free PBS
DMEM: Dulbecco’s modified essential medium
DMMB: dimethyl-methylene blue
dNTP: deoxy nucleotide triphosphate
ECM: extracellular matrix
EDTA: ethylene diamine tetra-acetic acid
ERK: extracellular signal-regulated kinase
ESC: embryonic stem cell
FABP: fatty acid binding protein
FCS: foetal calf serum
FGF: fibroblast growth factor
GDF: growth and differentiation factor
HD: high density
HDC: high density culture
HE: haematoxylin-eosin
hMSC: human mesenchymal stem cell
IGF: insulin-like growth factor
MAPK: mitogen activated protein kinase
N-CAM: neuronal cell adhesion molecule
OC: osteocalcin
OP: osteopontin
Osx: osterix
PBS: phosphate buffered saline
PPAR gamma: peroxisome proliferator-activated receptor gamma
RT-PCR: reverse transcription followed by polymerase chain reaction
TGF-β: transforming growth factor beta
