# Supplementary material for: Comparative Analysis of Osteogenic/Chondrogenic Differentiation Potential in Primary Limb Bud-Derived and C3H10T1/2 Cell Line-Based Mouse Micromass Cultures
Source: Int J Mol Sci. 2013 Aug 5;14(8):16141–67. doi: 10.3390/ijms140816141 (PMC3759904; doi:10.3390/ijms140816141)
Supplement: Supplementary file 1 [file ijms-14-16141-s001.pdf]

Supplementary Information

The conventional PCR reactions presented throughout the manuscript were performed in 3 independent experiments, but only gel images that represent the tendencies of mRNA expression patterns are shown.

Here, we present the mean values of relative optical densities as determined by ImageJ 1.46 freeware for all PCRs performed. In all cases, optical densities were normalised to the value of the earliest day of culturing where signals first appeared. Average values of relative optical densities were calculated and plotted against culturing days. Error bars represent standard deviations (S.D.) of these values.

Figure S1. Graphs representing mean values of relative optical densities of PCR results of chondrogenic marker genes.

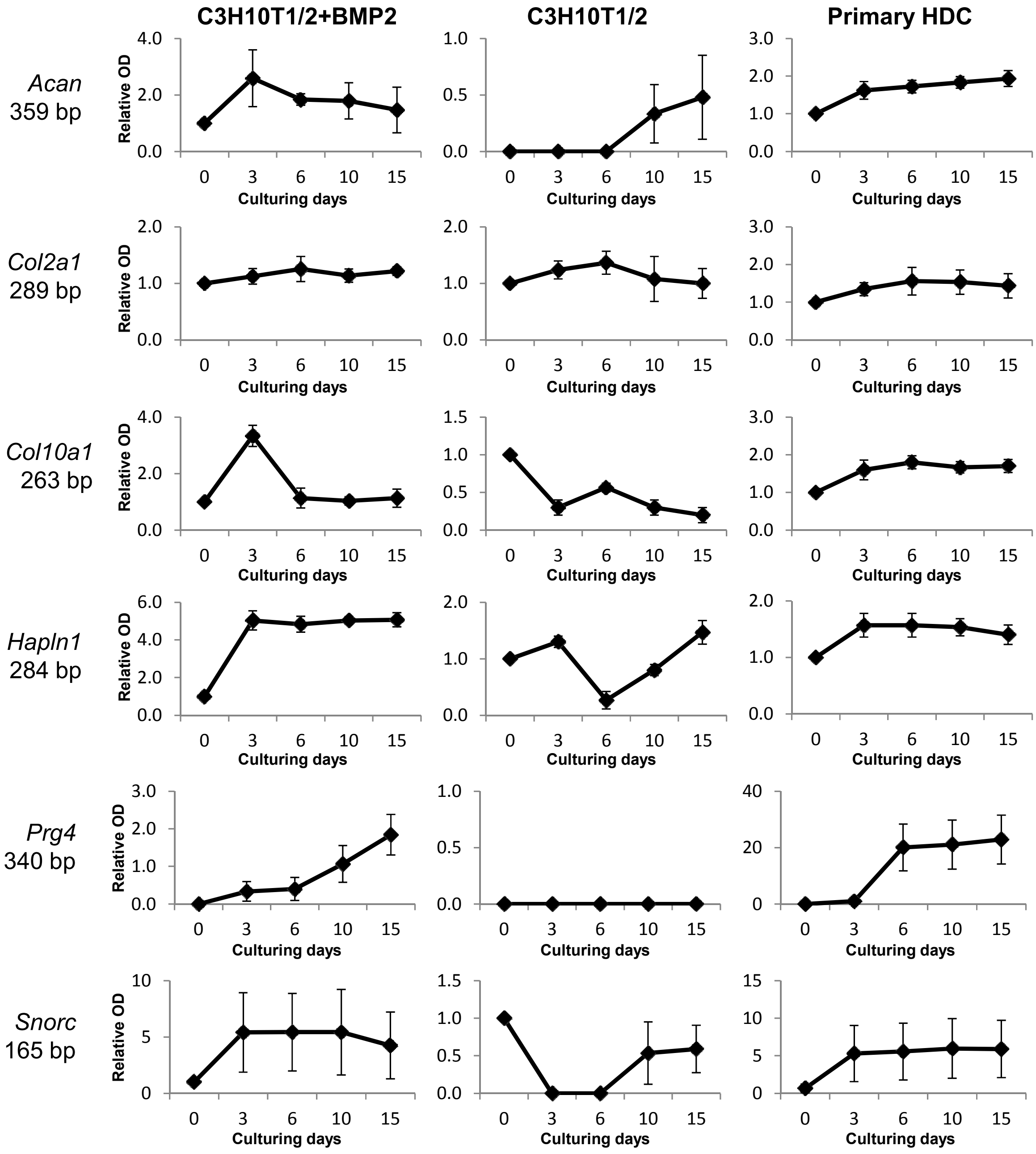

Figure S1. *Cont.*

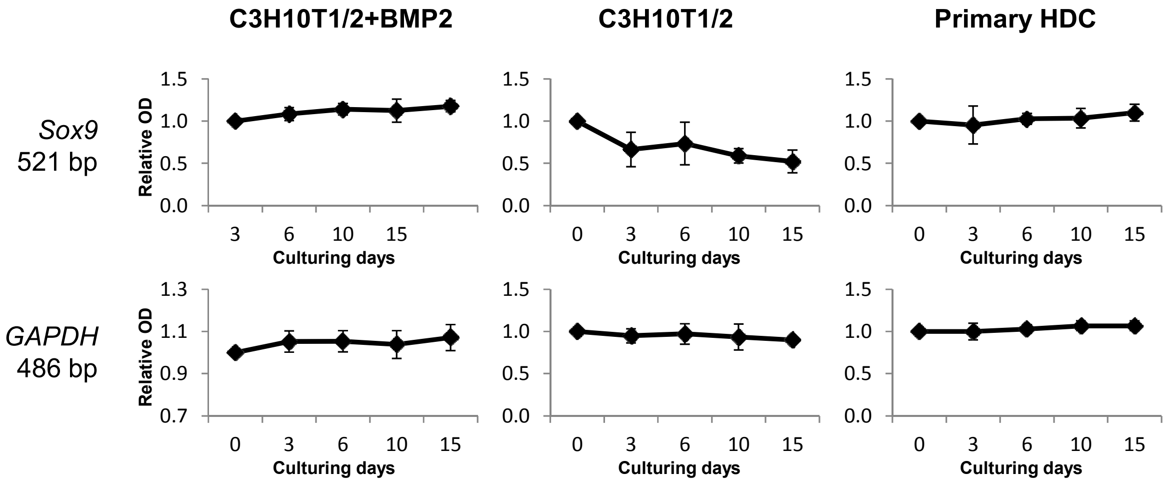

Figure S2. Graphs representing mean values of relative optical densities of PCR results of osteogenic marker genes.

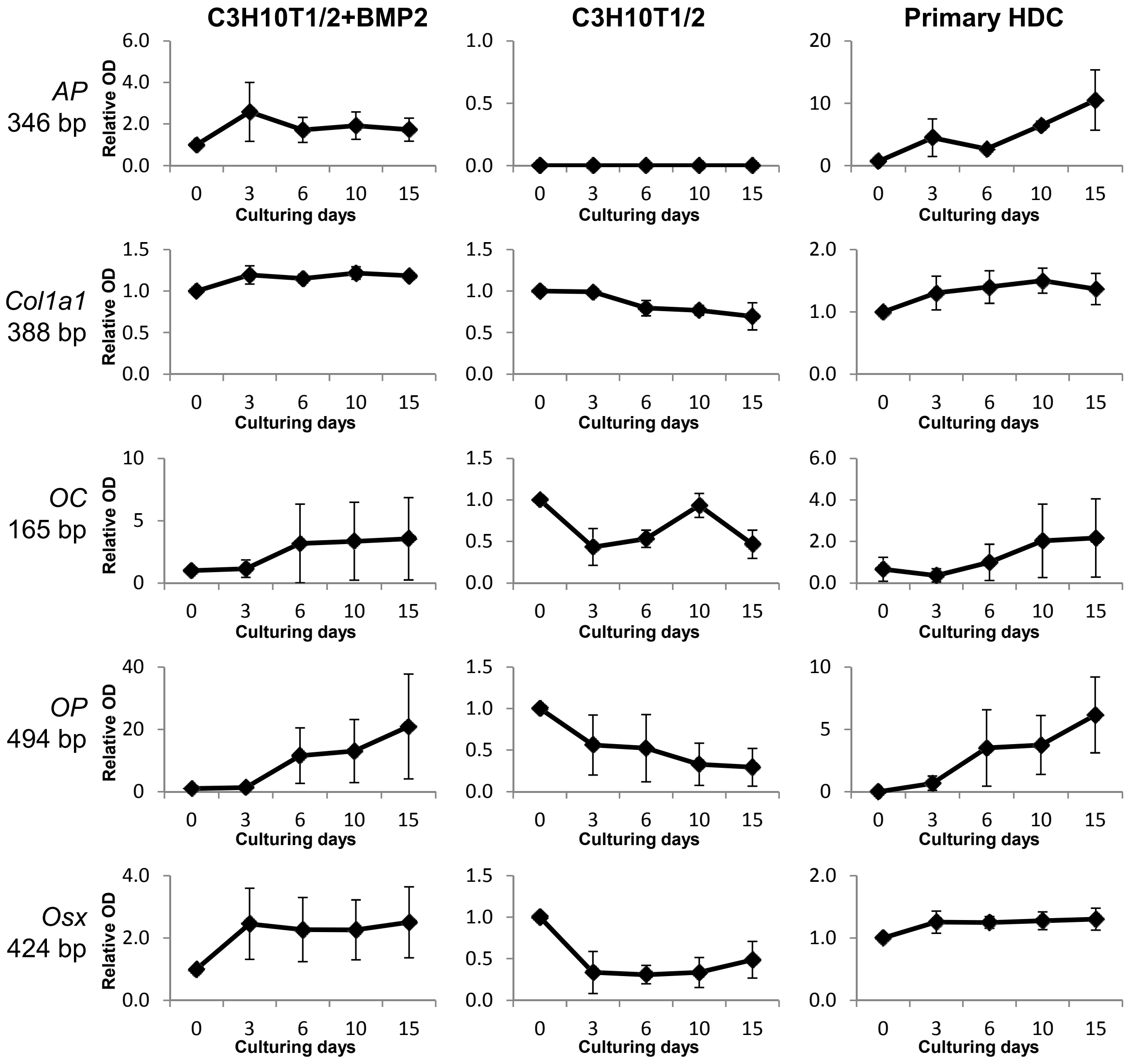

Figure S2. *Cont.*

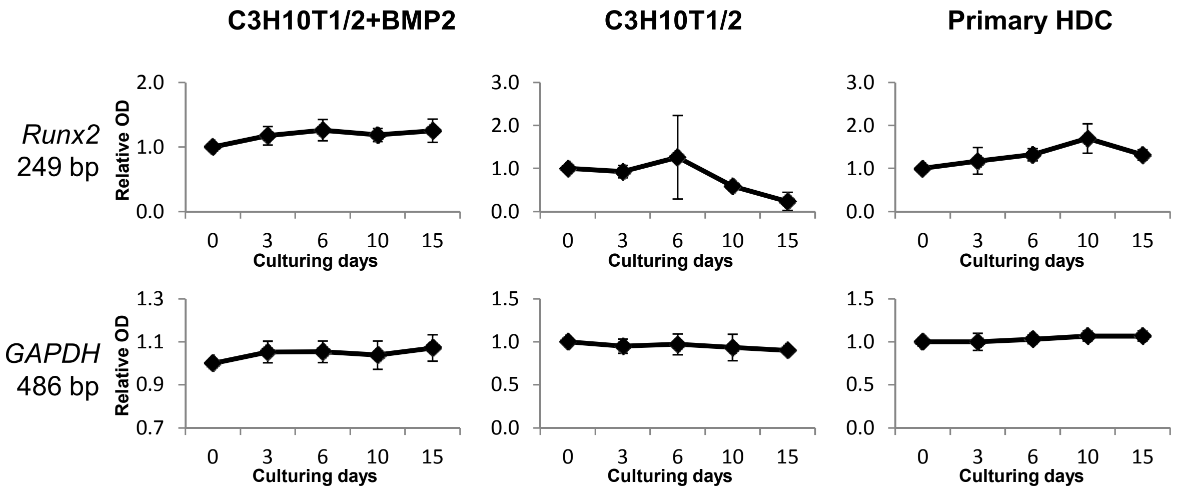

Figure S3. Graphs representing mean values of relative optical densities of PCR results of adipogenic marker genes.

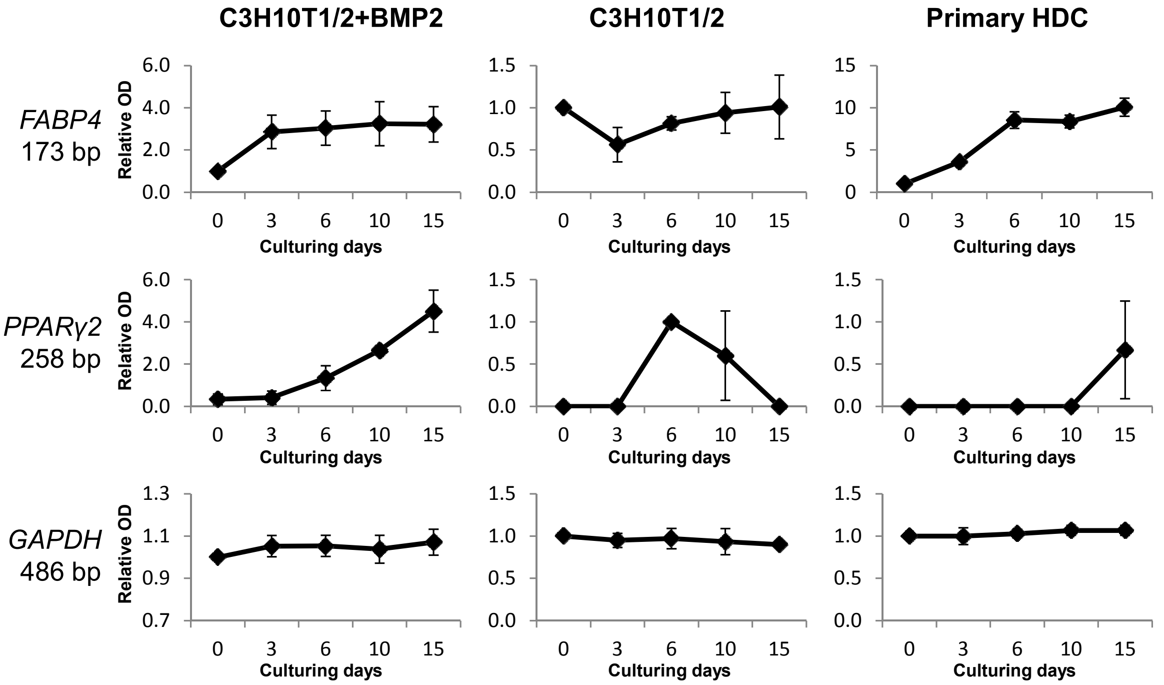

Figure S4. Graphs representing mean values of relative optical densities of PCR results of pluripotency factors.

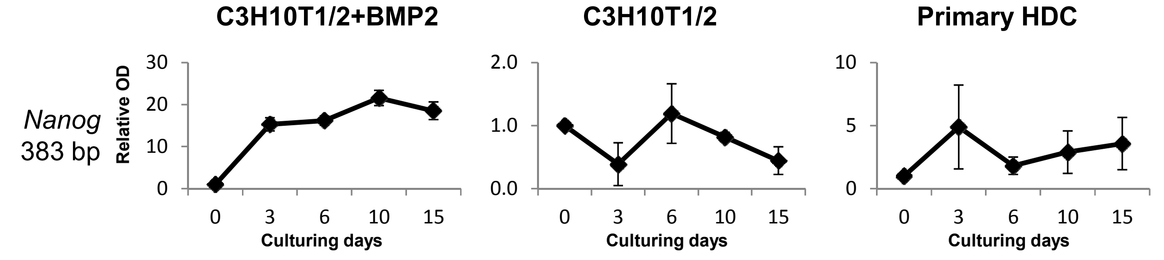

Figure S4. *Cont.*

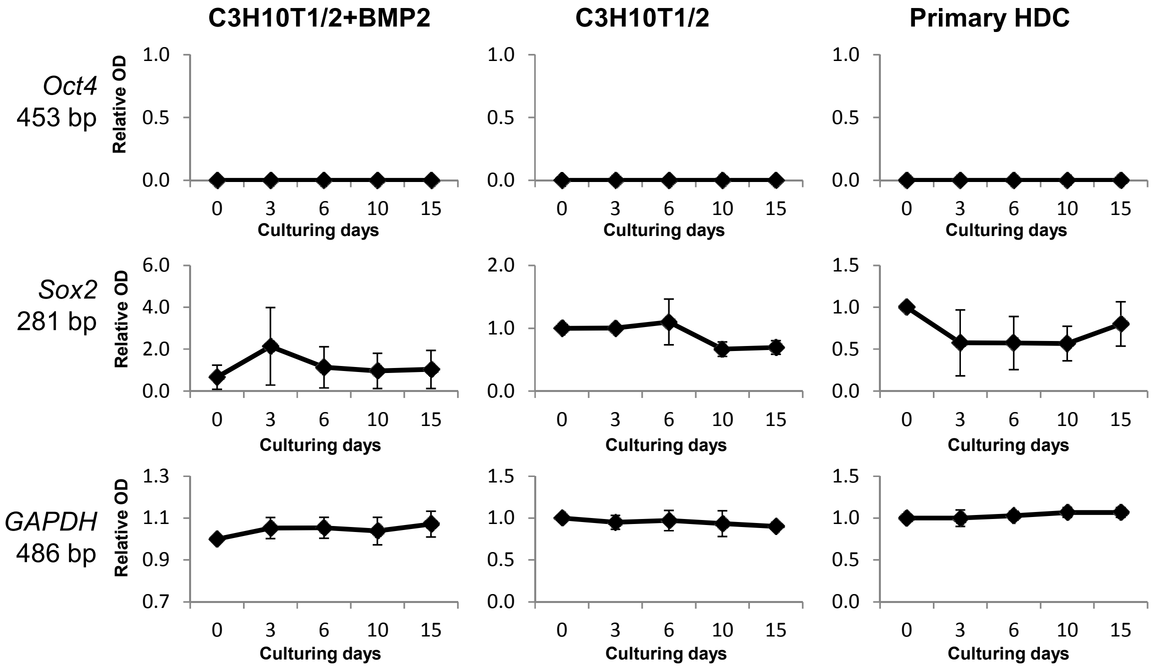

© 2013 by the authors; licensee MDPI, Basel, Switzerland. This article is an open access article distributed under the terms and conditions of the Creative Commons Attribution license (http://creativecommons.org/licenses/by/3.0/).
